# Ocular Vaccinia Infection in Dairy Worker, Brazil

**DOI:** 10.3201/eid2401.170430

**Published:** 2018-01

**Authors:** Maurício Teixeira Lima, Graziele Pereira Oliveira, Felipe Lopes Assis, Danilo Bretas de Oliveira, Sidiner Mesquita Vaz, Giliane de Souza Trindade, Jônatas Santos Abrahão, Erna Geessien Kroon

**Affiliations:** Universidade Federal de Minas Gerais, Belo Horizonte, Brazil (M.T. Lima, G.P. Oliveira, F.L. Assis, D.B. de Oliveira, G. de Souza Trindade, J.S. Abrahão, E.G. Kroon);; Casa de Caridade de Carangola, Carangola, Brazil (S.M. Vaz);; Federal dos Vales do Jequitinhonha e Mucuri, Diamantina, Diamantina, Brazil (D.B. de Oliveira)

**Keywords:** zoonoses, vaccinia virus, ocular infections, ocular vaccinia, viruses, occupational diseases, dairy worker, cattle, Brazil

## Abstract

We studied a clinical case of vaccinia virus that caused an ocular manifestation in a dairy worker in Brazil. Biologic and molecular analyses identified a co-infection with 2 isolates from different Brazilian vaccinia virus phylogenetic groups.

Detection of co-infections for various viral pathogens recently has increased (*1*,*2*). However, the presence of >2 viral etiologic agents often is not considered (*1*). The range of pathogens that can present co-infections and the association of these infections with the occurrence and severity of disease remain unclear (*1*,*2*). Vaccinia virus (VACV), the prototype virus of the genus *Orthopoxvirus*, has been associated with exanthematic outbreaks in Asia and South America that affect mainly dairy cattle and rural workers (*3*,*4*). In Brazil, several Brazilian VACV (VACV-BR) have been isolated and characterized biologically and phylogenetically. These studies demonstrated that circulating viruses belonged to at least 2 distinct genetic clusters (*4*–*8*). Previous studies have demonstrated the co-circulation of distinct VACV isolates during the same outbreak and VACV co-infecting horses and cattle (*6*–*8*).

We obtained 2 distinct VACV isolates from the same clinical sample from 1 eye of a rural worker. Our data show that the eye was co-infected with 2 VACV and demonstrates the detection and isolation of VACV from a natural case of ocular vaccinia infection.

In September 2015, an unvaccinated 45-year-old man who worked on a farm in Carangola County, Minas Gerais State, Brazil (20°44′06″S, 42°01′52″W), showed development of typical manifestations of vaccinia infection, including fever and painful vesiculopustular lesions (Technical Appendix Figure, panel A). He had lesions on the left hand, right arm, and nose and an atypical manifestation in the left eye with aches in the ocular globe and periorbital region. The clinical condition progressed to major visual acuity losses in the affected eye. He reported recent contact with sick cows on the farm during milking.

Dried swab specimens from his lesions were soaked in 200 μL phosphate-buffered saline containing amphotericin B (4 μg/mL), penicillin (200 U/mL), and streptomycin (100 μg/mL); homogenized; and centrifuged at 3,000 × *g* for 5 min (*4*). The supernatants were used for molecular diagnosis using orthopoxvirus-specific PCR that targeted the C11R gene, which encodes viral growth factor, and the A56R gene, which encodes viral hemagglutinin protein (*4*). All samples were positive for both orthopoxvirus targets.

Vero cells were cultured in 25-cm^2^ culture flasks and infected with the specimen supernatants to isolate the virus at 37°C until a cytopathic effect was detected (*4*). VACV was isolated from the hand, nose, and eye samples. These isolates were tested for their plaque phenotypes in BSC-40 cells incubated at 37°C for 48 h (*4*), which demonstrated the presence of at least 2 types of viral populations comprising small and large plaques in an estimated ratio 2:1. Two viral plaques (1 forming large and 1 forming small plaques) were obtained from the eye sample after 3 additional rounds of plaque purification in BSC-40 cells (Technical Appendix Figure, panel B) (*4*). The viral plaques were propagated and titrated by plaque assay in Vero cells, and their DNA was extracted (*4*).

We obtained the complete genomes using the Illumina MiSeq instrument (Illumina, San Diego, CA, USA) with the paired-end application. The sequence reads were assembled de novo using ABYSS software (http://www.bcgsc.ca/platform/bioinfo/software/abyss), and the resulting contigs were ordered by the python-based CONTIGuator.py software (http://contiguator.sourceforge.net). The GenBank accession numbers are MG012795 (small) and MG012796 (large).

Analysis of the complete genome revealed 92% similarity between the 2 isolates, and some genes confirm a remarkable variability (Technical Appendix Figure, panel C). We constructed a phylogenetic tree (Technical Appendix Figure, panel D) using the A56R gene sequence by the maximum-likelihood method and 1,000 bootstrap replicates in MEGA 6.02 (http://www.megasoftware.net). The analysis demonstrated a co-infection with viruses from both VACV-BR groups, such that the large-plaque clone clustered with group 2 VACV-BR isolates and the small-plaque clone clustered with group 1 VACV-BR isolates. We named these isolates Carangola eye virus 1 (small) and Carangola eye virus 2 (large).

Our study demonstrated the genetic and phenotypic variability between 2 viruses isolated from the same sample in a natural human co-infection with VACV. The viruses belong to 2 distinct VACV-BR groups, reinforcing and expanding previous work with other hosts (*6*–*8*). These results raise new questions about how co-infections with these viruses might change the aspects of an infection and its signs and symptoms, such as development of ocular vaccinia. Although cases of ocular vaccinia have been reported after vaccination and accidental laboratory infection (*9*,*10*), we proved the association and isolate VACV samples from a natural ocular vaccinia infection. The effort to understand singular aspects of VACV-BR co-infections should be increased, and further molecular and biologic characterizations of these samples should be conducted to identify and better understand the natural dynamics and signs and symptoms caused by VACV-BR.

Technical Appendix Ocular vaccinia infection in a 45-year-old male dairy farm worker, Brazil, 2015.
