# Supplementary material for: Ocular Vaccinia Infection in Dairy Worker, Brazil
Source: Emerg Infect Dis. 2018 Jan;24(1):161–2. doi: 10.3201/eid2401.170430 (PMC5749457; doi:10.3201/eid2401.170430)
Supplement: Technical Appendix — Ocular vaccinia infection in a 45-year-old male dairy farm worker, Brazil, 2015. [file 17-0430-Techapp-s1.pdf]

Article DOI: https://doi.org/10.3201/eid2401.170430

Ocular Vaccinia Infection in Dairy Worker

Technical Appendix

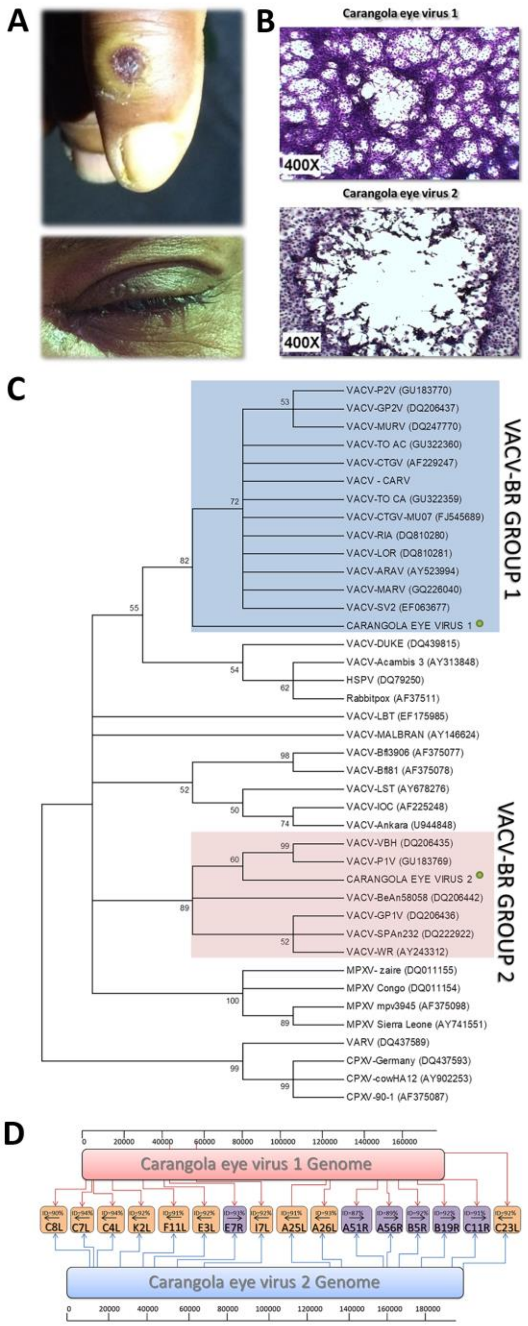

Technical Appendix Figure. Ocular vaccinia infection in a 45-year-old male dairy farm worker, Brazil, 2015. A) Lesion on the hand. B) Optical microscopy images of plaque phenotypes showing the lysis

plates of Carangola eye viruses 1 and 2 in BSC-40 cell monolayers stained with crystal violet. Original magnification 400×. C) Genome draft of Carangola eye viruses 1 and 2 shown some genes with nucleotide deletions and substitutions that differentiate the isolates. The percentage of identity is shown. D) Phylogenetic analysis using the maximum-likelihood method based on orthopoxvirus hemagglutinin genes. The tree was constructed with 1,000 bootstrap replicates using the Tamura-Nei model in MEGA 6.06 (http://www.megasoftware.net/). Numbers along branches are bootstrap values. Green circles indicate the vaccinia virus strains isolated in this study. Red and blue brackets indicate isolates belonging to groups 1 and 2, respectively, of the Brazilian vaccinia virus taxonomy. Cutoff value is *50%.*
